# Primary Low‐Grade Fibromyxoid Sarcoma of the Left Upper Lobe Treated With Uniportal Video‐Assisted Thoracoscopic Lobectomy

**DOI:** 10.1111/1759-7714.70391

**Published:** 2026-09-17

**Authors:** Giovanni Tacchi, Luca Frasca, Filippo Longo, Ilaria Suriano, Antonio Sarubbi, Lorenzo Nibid, Vincenzo Canalella, Pierfilippo Crucitti

**Affiliations:** ^1^ Thoracic Surgery Unit Fondazione Policlinico Universitario Campus Bio‐Medico Rome Italy; ^2^ PhD Course in Microbiology, Immunology, Infectious Diseases and Transplants (MIMIT) University Tor Vergata Rome Italy; ^3^ Master's Degree Program in Medicine and Surgery Campus Bio‐Medico University Rome Italy; ^4^ Anatomical Pathology Department Fondazione Policlinico Universitario Campus Bio‐Medico Rome Italy

**Keywords:** low‐grade fibromyxoid sarcoma, lung, uniportal VATS lobectomy

## Abstract

Low‐grade fibromyxoid sarcoma (LGFMS) is a rare tumor that usually affects young adults and arises on the proximal extremities and trunk. Primary pulmonary LGFMS is an extremely rare entity. We reported a postsurgical diagnosis of a primary lung LGFMS in a patient with single pulmonary nodule located in the left upper lobe. A uniportal thoracoscopic lobectomy with mediastinal lymph node dissection was performed. The histopathological report supposed the hypothesis of a primary lung LGFMS. The immunohistochemical investigation yielded intense and diffuse positivity of the neoplastic cells for MUC‐4. Next Generation Sequencing analysis showed the presence of EWSR1‐CREB3L2 gene fusion that was previously reported in literature only in a single case. Histological and molecular diagnosis for LGFMS is challenging and therefore a dedicated pathologist opinion is warranted. Surgery is the gold‐standard treatment for primary lung LGFMS and this is the first report about uniportal video‐assisted thoracoscopic lobectomy performed for such disease.

## Graphical abstract

Surgical excision is the cornerstone of treatment for low‐grade fibromyxoid sarcoma (LGFMS). Primary lung LGFMS has been described very rarely in the literature. To the best of our knowledge, this is the first report about uniportal video‐assisted thoracoscopic lobectomy performed for a primary lung LGFMS.

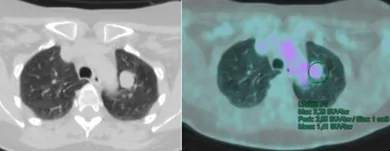

## 1 Introduction

Low‐grade fibromyxoid sarcoma (LGFMS) is a rare tumor that was first described by Evans in 1987 [1]. It usually affects young adults, and it is mainly located on the proximal extremities and trunk [2, 3]. Lungs are the main location for metastasis from LGFMS while primary pulmonary LGFMS is an extremely rare entity and only few cases have been previously reported in literature [4, 5, 6, 7, 8, 9, 10, 11]. Previous reports about primary lung LGFMS are listed in Table 1. Given the low mitotic rate, this tumor is poorly sensitive to radiotherapy and chemotherapy; therefore, surgery is the gold standard of treatment when feasible [12]. In this paper, we described a primary lung LGFMS resected with a uniportal video‐assisted thoracoscopic (VATS) lobectomy.

**TABLE 1:** Previous literature reports about primary lung LGFMS.

Authors	Years	Age/Sex	Size/Side	Treatments	Follow‐up (months)/local recurrence
Magro et al.	1998	20/F	2 cm, —	—	12/No
Kim et al.	2007	50/F	7.5 cm, Left	Lobectomy	—
Kurul et al.	2012	16/F	10x8 cm, Left	Lobectomy	24/No
Novikov et al.	2020	41/M	27 × 22 cm, Right	Lobectomy	—
Yoshimura et al.	2021	22/M	4 cm, Right	Lobectomy	—
Ershadi et al.	2021	26/F	15.5 × 13.5 cm/Left	Pneumonectomy	6/No
Ayadi et al.	2023	21/F	6 cm/Right	Lobectomy	6/No
Watanabe et al.	2024	22/M	8 cm/Left	Wedge resection	6/No
Zhang et al.	2024	39/F	7 × 5.6 × 5.3 cm/Left	Chemiotherapy + Immunotherapy (TP53 mutation)	—

*Note:* LGFMS, low‐grade fibromyxoid sarcoma; F, female; M, male.

## 2 Case Report

**FIGURE 1:**
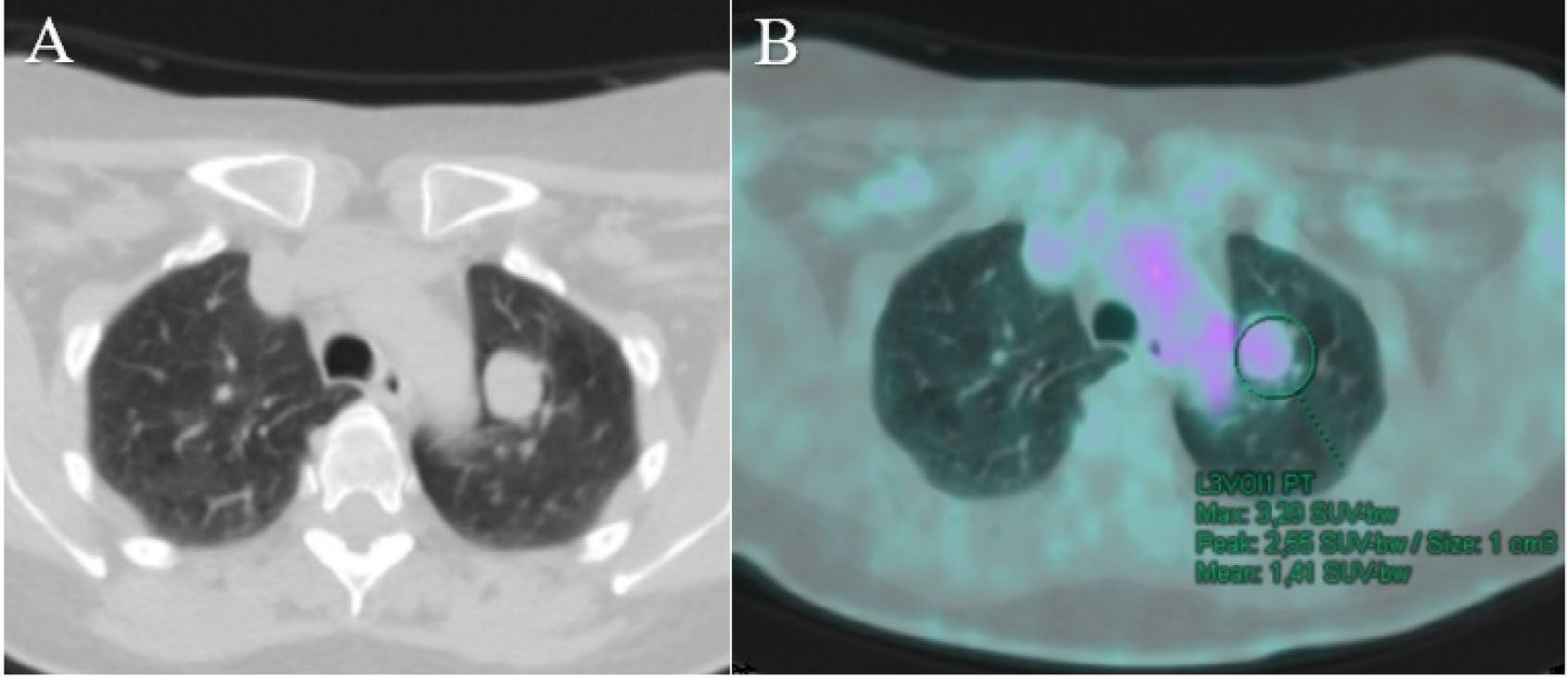
Section of the tumor on CT (A); section of the tumor on PET (B).

An asymptomatic 70‐year‐old woman was referred to our center because of incidental pulmonary nodule finding. She underwent a thoracic X‐ray for unrelated reason and then a computed tomography (CT) showing a 2.5 cm lesion located in the left upper lobe (Figure 1A). She was a former smoker with no other significant comorbidities. She had no previous surgical and oncological history, in particular any soft‐tissue tumor. In the suspicion of primary lung cancer, we prescribed an 18F‐fluorodeoxyglucose (18F‐FDG) positron emission tomography (PET) to complete the clinical and radiological staging. The exam yielded a single partial uptake of FDG from the nodule with a maximum standardized uptake value (SUV) of 3.29 without any focal uptake in mediastinal or hylar lymph nodes (Figure 1B).

**FIGURE 2:**
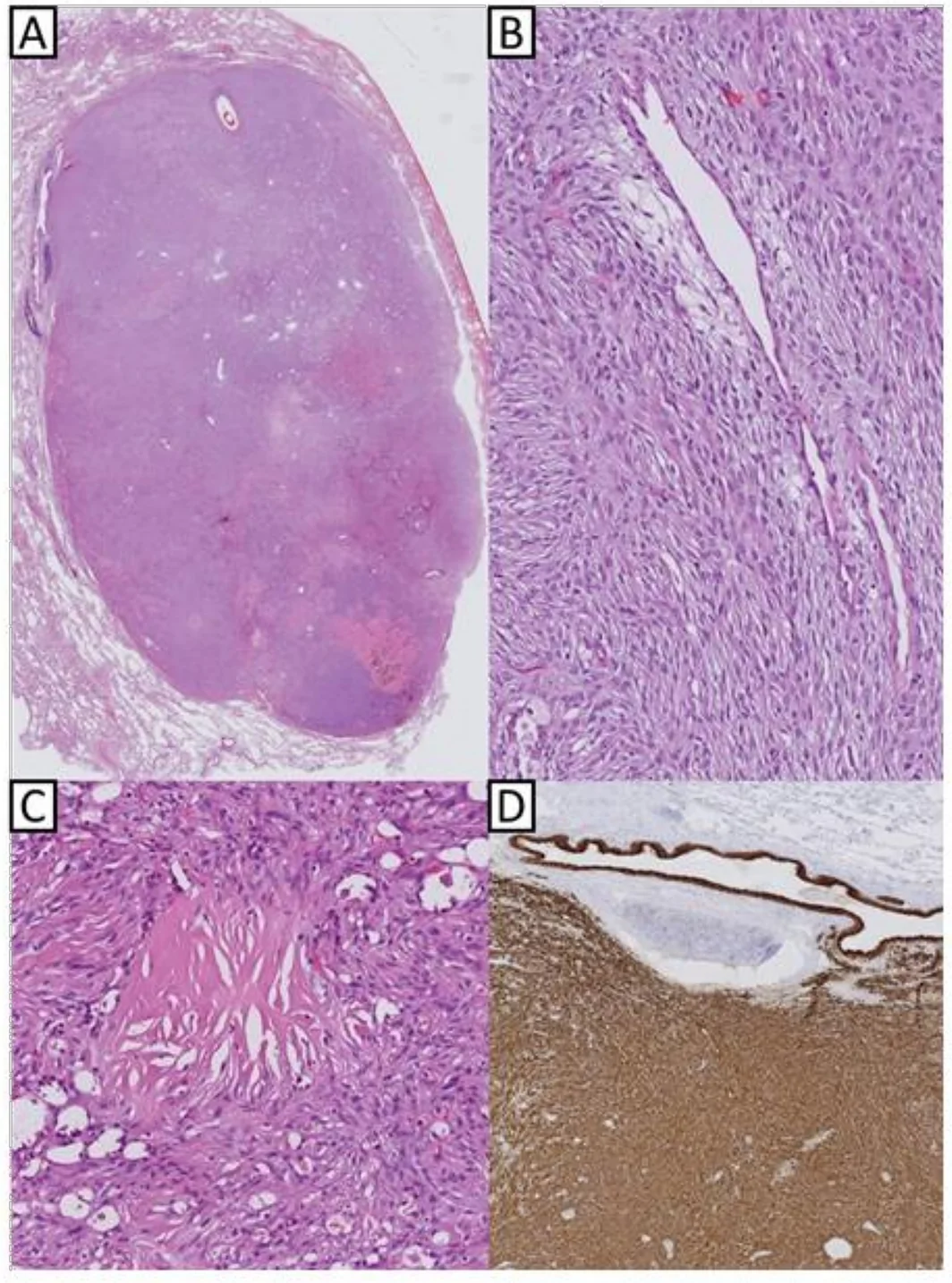
Histopathological findings from the surgical specimen. (A) Low‐power overview showing the well‐circumscribed lesion with pushing borders and a sharply defined margin (H&E, ×2). (B) Higher‐power view demonstrating a monotonous spindle‐cell proliferation within a focally myxoid stroma and featuring branching staghorn‐type vessels (H&E, ×20). (C) Higher‐power view highlighting hyalinized pseudorosettes and entrapped pneumocytes within the tumor (H&E, ×20). (D) MUC4 immunohistochemical staining showing diffuse positivity in tumor cells; respiratory epithelium and pneumocytes serves as a positive and negative internal control respectively (×10).

We submitted the patient to a CT‐guided biopsy which was performed about 1 month before the surgical operation. Although there were some cellular atypies, no definitive diagnosis was obtained. Given the malignant suspect deriving from the biopsy, the tumor dimension and location, after obtaining the informed consent from the patient, we performed a uniportal thoracoscopic left upper lobectomy. We made a 4 cm skin incision at fifth left intercostal space and accessed the pleural cavity via a soft tissue retractor. Our dissection strategy followed the Gonzalez‐Rivas technique and we used customized surgical instruments that allowed us to carry out the operation without significant fencing. We did not face any intraoperative challenges related to the tumor. A frozen section analysis was performed on the entire specimen after lobectomy completion in order to decide whether to carry out a lymphadenectomy, given the possibility of postoperative complications related to this procedure. This analysis yielded a malignant diagnosis without discriminating between epithelial tumor or other variant; therefore, we performed mediastinal and hilar lymphadenectomy. The postoperative course was uneventful and the patient was discharged at home on the third postoperative day. Here is the pathologic report:

- “MACROSCOPIC: A lung lobe measuring 21 × 7.5 × 2.5 cm, with a pulmonary resection margin 7 cm in length and a bronchial resection margin 1.7 cm in length. The pleural surface stains uniformly. On sectioning, a subpleural parenchymal nodule is found, solid, with a maximum size of 2.5 cm, regular margins, hard, whitish, located 2.2 cm from the bronchial margin and 3.5 cm from the pulmonary margin.
- MICROSCOPIC (Figure 2): Pulmonary parenchyma and pleural surface site of spindle and oval cell proliferation, monomorphic and arranged in sometimes vorticoid bundles that entrap alveolar and bronchiolar structures. Proliferation alternates between hypo and hypercellular areas in the context of partly sclerotic and only focally myxoid stroma with occasional hyaline pseudorosette formation. Hemangiopericytoma‐like vessels coexist. The immunohistochemical investigation documented intense and diffuse positivity of the neoplastic cells for MUC‐4 and cyclin‐D1, focal and weak positivity for HEMA, positivity in rare neoplastic elements for beta‐catenin, focal and non‐specific positivity for CD163 and negativity for CK‐AE1/AE3, CALRETININ, STAT6, SS18‐SSX, CD34, CD117, DOG1, SMA, DESMINE, PGR, $100, SOX10, CD31, ERG, and ALK (D5F3). A retained nuclear expression of INI1 was found. The proliferative index was < 1 mitosis per 10 high‐power fields. Histopathological picture suspected in first hypothesis for low‐grade fibromyxoid sarcoma.

## 3 Discussion

LGFMS, generally known as Evans tumor, usually occurs in the proximal extremities (45.1%) or pelvis (18.6%), followed by the chest wall (13.7%), retroperitoneum (8.8%), abdomen (7.8%) and head or neck (5.9%) [13]. LGFMS rarely arises from the lung. In our case, given the clinical and radiological history, we first suspected non‐small cell lung cancer (NCSLC). Indeed, the patient had no previous oncologic history and preoperative imaging showed no evidence of any extrathoracic lesions. After evaluation at our weekly scheduled multidisciplinary meeting [14, 15], a left upper lobectomy with mediastinal lymph node dissection was performed. We did not carry out a wedge resection because of the malignant suspect deriving from the biopsy, the tumor dimension and its paraortic location. Performing uniportal VATS lobectomy facilitated a fast‐track pathway for our patient because of the advantages reported in literature regarding uniportal VATS (e.g., reduction in post‐operative pain and minor/major complications, better recovery of physical function, etc.) [16]. Globally we had a similar diagnostic approach to most of previous reports with both CT and PET‐CT scan carried out in the pre‐operative setting. This item was linked to the small dimension of the target lesion so that it was simple to perform a minimally invasive procedure. In addition, our patient was significantly older than other reports and this an other unique aspect of our case. Our case confirms how preoperative diagnosis of primary pulmonary LGFMS may be challenging. Histologically, LGFMS presents as spindle‐shaped cells with fibrous and myxoid areas. The heterogeneous histological findings hinder the differential diagnosis with soft tissue tumors (e.g., solitary fibrous tumor, malignant peripheral nerve sheath tumors, myxoid liposarcoma, myxofibrosarcoma). Immunohistology analysis could be very useful as in most cases it is positive for Mucin 4 (MUC4) [17]. Doyle et al. reported 49 resected LGFMS cases with 100% showed positivity for MUC4 [18]. For what concerns genetics, this tumor is characterized by chromosomal translocations involving the Fused in Sarcoma (FUS) gene and cAMP responsive element‐binding protein 3‐like 2 and like 1 (FUS‐CREB3L2 and FUS‐CREB3L1). The FUS‐CREB3L2 translocation is more common (75%–95%) than FUS‐CREB3L1 (5%–10%) [19, 20]. In the present case, immunohistology showed diffuse positivity for MUC4 but there were no FUS translocations. Although the immunophenotype was highly suggestive of LGFMS, Next Generation Sequencing (NGS) was pursued to obtain definitive molecular confirmation and to exclude histologic mimics. The NGS analysis was performed on FFPE tumor tissue using the Archer FusionPlex Sarcoma v2 panel on an Ion Torrent/IonGeneStudio S5 Prime platform. The assay identified an EWSR1::CREB3L2 fusion transcript involving EWSR1 exon 8 as the 5′ partner and CREB3L2 exon 5 as the 3′ partner. The reference transcripts were EWSR1 NM_005243.4 and CREB3L2 NM_194071.4. The fusion was reported as in‐frame, with 54 supporting reads. The genomic breakpoint coordinates were chr22:29684775 for EWSR1 and chr7:137593126 for CREB3L2, according to GRCh37/hg19. The quality‐control metric was adequate, with a reported QC value of 98.5, above the laboratory threshold of ≥ 10; total fragments were 989 689, reads after trimming were 868 467, and RNA‐associated reads were 652 641. This is worth mentioning as the EWSR1::CREB3L2 fusion has been only described in a previous report of a pure LGFMS arising in the parotid gland [21]. EWSR1::CREB3L2 fusions may also occur in sclerosing epithelioid fibrosarcoma (SEF), but any conventional SEF‐like or hybrid LGFMS/SEF morphology was identified after examination of the entire tumor. Fluorescence in situ hybridization (FISH) analysis for FUS rearrangement was not performed as comprehensive RNA‐based NGS was considered sufficient to evaluate known and alternative gene fusions associated with LGFMS. The case was further discussed at our multidisciplinary meeting where no adjuvant treatment was suggested and the patient was sent to follow‐up based on contrast enhanced total body CT performed every 3 months. Nowadays there is no sign of radiological or clinical recurrence.

In the light of what is reported, referral to a dedicated soft‐tissue pathologist is crucial for an accurate diagnosis. In conclusion, we reported an incidental post‐surgical diagnosis of a primary lung LGFMS, an extremely rare entity. Preoperative and postoperative diagnosis is demanding; therefore, an expert pathologist opinion is recommended. Surgical excision is the mainstay of treatment, and to the best of our knowledge, this is the first report in literature about uniportal VATS lobectomy performed for a primary lung LGFMS.

## Author Contributions

**Giovanni Tacchi:** writing – original draft, writing – review and editing. **Luca Frasca:** conceptualization, methodology, data curation. **Filippo Longo:** validation. **Ilaria Suriano:** investigation. **Antonio Sarubbi:** visualization. **Lorenzo Nibid:** methodology. **Vincenzo Canalella:** resources. **Pierfilippo Crucitti:** supervision, project administration.

## Funding

The authors have nothing to report.

## Conflicts of Interest

The authors declare no conflicts of interest.

## Data Availability

The data that support the findings of this study are available on request from the corresponding author. The data are not publicly available due to privacy or ethical restrictions.
